# Effect of ranibizumab combined with laser photocoagulation in the treatment of diabetic macular edema

**DOI:** 10.12669/pjms.40.6.9213

**Published:** 2024-07

**Authors:** Junhua Hu, Hongfei Liao, Houli Xia, Yinglin Liao, Dan Yuan

**Affiliations:** 1 Junhua Hu, Department of Ophthalmology, The Affiliated Eye Hospital of Nanchang University, 463 Bayi Avenue, Nanchang, Jiangxi Province 330000, P.R. China; 2 Hongfei Liao, Department of Ophthalmology, The Affiliated Eye Hospital of Nanchang University, 463 Bayi Avenue, Nanchang, Jiangxi Province 330000, P.R. China; 3 Houli Xia, Department of Ophthalmology, The Affiliated Eye Hospital of Nanchang University, 463 Bayi Avenue, Nanchang, Jiangxi Province 330000, P.R. China; 4 Yinglin Liao, Department of Ophthalmology, The Affiliated Eye Hospital of Nanchang University, 463 Bayi Avenue, Nanchang, Jiangxi Province 330000, P.R. China; 5 Dan Yuan, Department of Ophthalmology, The Affiliated Eye Hospital of Nanchang University, 463 Bayi Avenue, Nanchang, Jiangxi Province 330000, P.R. China

**Keywords:** Ranibizumab, Diabetic macular edema, Laser photocoagulation

## Abstract

**Objective:**

To explore the clinical effect of ranibizumab combined with laser photocoagulation (LP) in treating diabetic macular edema (DME).

**Methods:**

We retrospectively reviewed the clinical data of 118 patients with DME admitted to The Affiliated Eye Hospital of Nanchang University from May 2021 to March 2023. Among them, 38 patients received LP alone (Laser-group), 39 patients received ranibizumab alone (Ranibizumab-group), and 41 patients received LP combined with ranibizumab (Combined-group). The improvement of macular edema (ME), visual acuity, and complications between the groups were compared.

**Results:**

The time of ME regression, exudation absorption and fundus hemorrhage absorption in the Combined-group was shorter than in the Laser-group and the Ranibizumab-group (P<0.05). After treatment, the CMT and RNV of the three groups decreased compared to pretreatment levels and were lower in the Combined-group compared to the Laser-group and the Ranibizumab-group (P<0.05). BCVA increased after the treatment in all groups, and was markedly higher in the Combined-group than in the Laser and the Ranibizumab-groups (P<0.05). NO were higher in the Combined-group compared to the Laser-group and the Ranibizumab-group. The post-treatment VEGF levels decreased in all groups, and were significantly lower in the Combined-group compared to the Laser-group and the Ranibizumab-group (P<0.05). The incidence of complications in the Combined-group was lower than in the Laser-group and the Ranibizumab-group (P<0.05).

**Conclusions:**

Compared to ranibizumab or LP alone, ranibizumab combined with LP is more effective in reducing ME in patients with DEM, and is associated with fewer complications.

## INTRODUCTION

Diabetic retinopathy is a common complication of diabetes.^1^ In recent years, with the aging of the population, and changing lifestyle and dietary patterns, the incidence of diabetes and diabetic retinopathy has been on the rise.^1^,^2^ Diabetic macular edema (DME) is an important cause of visual impairment in diabetic patients,^3^ and delayed or ineffective intervention may lead to visual function loss. Therefore, timely implementation of safe and effective treatment for DME is of great significance.^3^,^4^

LP is considered the gold standard for DME and the only treatment that has been shown to be effective in eliminating retinal neovascularization.^5^ However, in some patients, retinal neovascularization may still progress even after laser treatment, and the overall outcome may fall short of clinical expectations.^5^,^6^ Study has found that vascular endothelial growth factor (VEGF) can stimulate angiogenesis, increase its permeability, and accelerate the progress of DME.^7^ Therefore, anti-VEGF drugs have emerged as potential agents to improve the therapeutic effect of DME.^7^,^8^ Ranibizumab is a second-generation humanized recombinant monoclonal antibody fragment against VEGF, which can selectively bind to VEGF and inhibit endothelial cell proliferation, thereby exerting therapeutic effects.^9^,^10^

Several studies have suggested the superiority of combining laser treatment with anti-VEGF therapy in patients with DME.^5^,^11^ However, most studies only compared the combined therapy with LP alone or ranibizumab alone, and few studies compared all three simultaneously. In recent years, our hospital has adopted the combination of ranibizumab and laser therapy for treating patients with DME. The aim of this study was to review and analyze the treatment status of the patients and to clarify the intervention effect of combination therapy with ranibizumab and laser therapy, providing practical reference for clinical treatment of DME.

## METHODS

We retrospectively selected clinical data of 118 patients with DME (69 were males and 49 were females) admitted to our hospital from May 2021 to March 2023. A total of 38 patients were treated with LP alone and assigned to the Laser-group; 39 patients were treated with ranibizumab alone and assigned to the Ranibizumab-group; 41 patients were treated with LP combined with ranibizumab and assigned to the Combined-group.

### Ethical Approval

The ethics committee of The Affiliated Eye Hospital of Nanchang University approved this study: No. RMYY20220527161, date: 2022-05-26.

### Inclusion criteria:

- Patients met DME diagnostic standards.^10^
- The diagnosis was confirmed by fundus fluorescein angiography and slit lamp microscopy.
- Age ≥ 18 years old.
- CMT ≥ 300 μm.
- Complete clinical data.
- Monocular onset.

### Exclusion criteria:

- Individuals with other eye diseases.
- Allergy to the study drug, allergy or lack of tolerance to the study protocol.
- Existence of immune system and blood system diseases.
- Patients who received anti-VEGF treatment before inclusion in the study.
- Individuals with contraindications for intraocular medication.

### Laser-group

Laser photocoagulation therapy was performed using Nd: YAG532 wavelength fundus laser instrument produced by French company Guangtai Medical. Before the treatment, fundus fluorescein angiography was routinely performed (HRA-2, Heidelberg, Germany). Compound topicamide eye drops (Medoli, Japan) were administered to dilate the pupils to 7-8mm. Surface anesthesia was performed using propacaine hydrochloride eye drops (Elkein, Belgium), and the intensity of the photocoagulation reaction was set according to the Tso’s grading method. For patients with extensive non-perfusion areas of retinal capillaries, or those with retinal neovascularization, extensive fluorescence leakage, microangioma formation, and lesion location greater than 1/4 quadrant, whole retinal photocoagulation was administered with specific parameters: power set to 150-450 mW, spot diameter of 100-200μm, exposure time of 0.1-0.2 seconds, Grade-III laser spot, once a week, a total of 1200-1600 points, completed in 4-5 times. Patients with localized retinal non-perfusion areas were treated with local photocoagulation in areas such as capillary non-perfusion areas and the surrounding retina. Specific parameters were as follows: power set to 200-400 mW, spot diameter of 150-200μm, exposure time of 0.1-0.2 seconds, Class-III laser spot. After the treatment, diclofenac sodium eye drops (Shenyang Xingqi Pharmaceutical Co., Ltd.) were given to the eyes four times a day for three days.

### Ranibizumab-group

Ranibizumab treatment performed was as follows: First, surface anesthesia was administered with tetracaine eye drops. The eyelid was opened with an eyelid opener, and the conjunctival sac was effectively cleaned with iodophor solution (Alcon, USA). The needle was inserted into the flat part of the ciliary body about 3.5 mm from the infratemporal part to the corneal margin (making sure that the needle tip is perpendicular to the scleral surface). When the needle was clearly inserted into the vitreous cavity, 0.05 ml of ranibizumab (Novartis Pharma Stein AG, Switzerland) was slowly injected into the vitreous cavity. Ofloxacin eye ointment (Japan Santen Pharmaceutical Company) was applied to the conjunctival sac to cover the affected eyes, and Tobramycin eye drops (American Alcon Company) were applied to the eyes two weeks after the operation.

### Combined-group

Patients in the combined group received ranibizumab combined with LP. The ranibizumab treatment was the same as the Ranibizumab-group. The patients received LP treatment 7-10 days after ranibizumab treatment and the process of LP treatment was the same as the LP-group.

### Outcome measures:

### Symptom improvement

It was noted when macular edema (ME) subsides, when exudation is absorbed, and when eyeground hemorrhage is absorbed. Central macular thickness (CMT), best corrected visual acuity (BCVA), and retinal neovascularization (RNV) levels were measured before treatment and six months after treatment. CMT was measured by Optical coherence tomography (Heidelberg, Germany); BCVA was measured by an international standard visual acuity chart; RNV was measured by fundus fluorescein angiography (HRA-2, Heidelberg, Germany).

### Biochemical indicators

NOS and VEGF levels were measured in the serum from 4 ml of fasting venous blood by enzyme-linked immunosorbent assay. The reagent kit was purchased from R&D Company in the United States and the incidence of complications.

### Statistical Analysis

The statistical software used was SPSS26.0 (IBM Corp, Armonk, NY, USA). The measurement data that met the normal distribution were expressed by mean ± standard deviation. Multiple group comparisons were conducted using one-way ANOVA, and pairwise comparisons were conducted using LSD test afterwards. Paired t-tests were used for intragroup comparison before and after treatment. The measurement data that did not meet the normal distribution were presented by M (IQR). Kruskal waills H-test was used for inter group comparison, and Nemenyi method was used for pairwise comparison. The Wilcoxon signed rank test was used for intra group comparison before and after treatment. Counting data were expressed by n (%), and Chi-squared test was used for comparison between groups. *P*<0.05 indicated statistical significance.

## RESULTS

Clinical data of a total of 118 patients with DME met the inclusion criteria. Of them, 38 patients were in the Laser-group, 39 in the Ranibizumab-group, and 41 patients in the Combined-group. The age of the patients ranged from 48 to 82 years, with an average age of 64.02 ± 8.94 years. In terms of the affected eye, 54 patients had ME in the left eye and 64 in the right eye. The course of diabetes was 4~22 years, with an average of 11.92 ± 4.26 years. There was no significant difference in baseline data among the three groups (*P*>0.05), Table-I.

**Table-I:** Comparison of baseline data among three groups.

Group	n	Gender (male/female)	Age (years)	Affected eye (left/right)	Course of diabetes (years)
Combined-group	41	26/15	63.22±9.35	20/21	10.90±4.13
Laser-group	38	22/16	64.92±8.85	19/19	12.42±4.00
Ranibizumab-group	39	21/18	63.97±8.73	15/24	12.49±4.55
*χ*^2^/*F*		0.761	0.354	1.263	1.799
*P*		0.683	0.703	0.532	0.170

The time of ME regression, exudation absorption and fundus hemorrhage absorption in the Combined-group was shorter than that in the Laser-group and the Ranibizumab-group (*P*<0.05). There was no significant difference in the time of ME regression, exudation absorption and fundus hemorrhage absorption between the Laser-group and the Ranibizumab-group (*P*>0.05), Table-II.

**Table-II:** Comparison of symptom improvement time among three groups (week).

Group	n	ME regression	Exudation absorption	Fundus hemorrhage absorption
Combined-group	41	4(3, 5)^ab^	10(9, 11)^ab^	2(2, 3)^ab^
Laser-group	38	5(4, 6)	12(11, 13)	3(3, 4)
Ranibizumab-group	39	5(5,6)	12(10, 13)	3(3, 4)
H		31.256	22.826	27.301
P		<0.001	<0.001	<0.001

***Note:*** Compared with the Laser-group, ^a^P<0.05; Compared with the Ranibizumab-group, ^b^P<0.05.

Before treatment, there was no significant difference in CMT, BCVA, and RNV between the three groups (*P*>0.05). After treatment, the CMT and RNV of the three groups decreased compared to pretreatment levels and was lower in the Combined-group compared to the Laser-group and the Ranibizumab-group (*P*<0.05). BCVA increased after the treatment in all groups, and was markedly higher in the Combined-group than in the Laser and the Ranibizumab-groups (*P*<0.05), Table-III

**Table-III:** Comparison of CMT, BCVA, and RNV between the three groups.

Time	Group	n	CMT (um)	BCVA	RNV (mm^2^)
Before treatment	Combined-group	41	530.56±36.05	0.4(0.3, 0.5)	23.88±4.45
	Laser-group	38	543.03±29.12	0.4(0.3, 0.5)	22.79±3.58
	Ranibizumab-group	39	536.26±29.64	0.3(0.3, 0.5)	24.05±3.29
	F/H		1.510	1.616	1.235
	P		0.225	0.446	0.295
After treatment	Combined-group	41	244.39±25.39^ab*^	0.8(0.7, 0.9)^ab*^	5.05±1.83^ab*^
	Laser-group	38	281.11±26.07^*^	0.6(0.5, 0.7)^*^	9.18±2.22^*^
	Ranibizumab-group	39	276.97±27.70^*^	0.6(0.5, 0.7)^*^	9.15±2.35^*^
	F/H		23.261	38.562	49.771
	P		<0.001	<0.001	<0.001

***Note:*** Compared with the Laser-group, ^a^P<0.05; Compared with the Ranibizumab-group, ^b^P<0.05; Compared with before treatment in this group,

* P<0.05.

Before treatment, levels of NOS and VEGF were comparable in the three groups (*P*>0.05). There was a significant increase in the levels of NOS in all groups after treatment, and the levels of NO were higher in the Combined-group compared to the Laser-group and the Ranibizumab-group. The post-treatment VEGF levels decreased in all groups, and were significantly lower in the Combined-group compared to the Laser-group and the Ranibizumab-group (*P*<0.05), Table-IV. The incidence of complications in the Combined-group (4.88%) was significantly lower than that in the Laser-group (21.05%) and the Ranibizumab-group (20.51%) (*P*<0.05), Table-V.

**Table-IV:** Comparison of biochemical indicators among three groups.

Time	Group	n	NOS (U/ml)	VEGF (pg/ml)
Before treatment	Combined-group	41	38.98±6.19	260.80±43.55
	Laser-group	38	37.63±8.11	263.66±45.66
	Ranibizumab-group	39	39.67±8.28	257.44±56.50
	F		0.721	0.157
	P		0.488	0.855
After treatment	Combined-group	41	107.83±9.19^ab*^	84.05±24.59^ab*^
	Laser-group	38	96.58±10.59^*^	123.55±20.92^*^
	Ranibizumab-group	39	97.15±11.63^*^	119.54±23.04^*^
	F		14.611	35.966
	P		<0.001	<0.001

***Note:*** Compared with the Laser-group, ^a^P<0.05; Compared with the Ranibizumab-group, ^b^P<0.05; Compared with before treatment in this group,

* P<0.05.

**Table-V:** Comparison of the incidence of complications among the three groups.

Group	n	Edema	Pupil injury	Color anomalopia	Increased intraocular pressure	Total occurrence rate
Combined-group	41	1 (2.44)	0 (0.00)	1 (2.44)	0 (0.00)	2 (4.88)^ab^
Laser-group	38	4 (10.53)	1 (2.63)	1 (2.63)	2 (5.26)	8 (21.05)
Ranibizumab-group	39	2 (5.13)	1 (2.56)	3 (7.69)	2 (5.13)	8 (20.51)

***Note:*** Compared with the Laser-group, ^a^P<0.05; Compared with the Ranibizumab-group, ^b^P<0.05.

## DISCUSSION

Our study showed that the combined ranibizumab/laser photocoagulation therapy has high application value and is superior to ranibizumab and laser monotherapies. Combined treatment is more effective in alleviating clinical symptoms, improving visual acuity, and reducing the occurrence of complications in patients with DME, which is basically consistent with Ishibashi et al^12^, Berger et al^13^, and Mitchell et al.^14^

Howaidy et al.^10^ used laser therapy and ranibizumab to treat patients with DME on the basis of routine intervention, and showed that it can effectively reduce the thickness of the macular fovea and prevent the occurrence of related complications, which is consistent with the results of our study. Ranibizumab has numerous advantages such as strong specificity, high bioavailability, and strong permeability, and can effectively inhibit VEGF in the retina, prevent vascular leakage, and reduce retinal edema.^10^ Antoszyk et al.^15^ used ranibizumab in patients with diabetes retinopathy with or without diabetes ME, and confirmed that it can improve patients’ vision and alleviate clinical symptoms. Kayıkçıoğlu et al.^16^ used a combination of subthreshold micro pulse laser and ranibizumab to treat patients with DME, and showed that it not only improves the treatment effect, but also reduces the risk of complications. The results of our study are consistent with these reports. In the combination therapy of laser and ranibizumab, injecting ranibizumab into the vitreous cavity can to some extent shrink retinal neovascularization, absorb the original glass volume of blood, and reduce tissue edema and vascular leakage. As a result, the retina is in a dry state, which provides ideal conditions for laser photocoagulation treatment, ensures good spot response under low laser energy state, and reduces the occurrence of bleeding and other related complications.^10^,^15^,^16^ Mi et al.^11^ also confirmed that the combination of ranibizumab and micro pulse laser therapy can reduce the number of anti-VEGF drug injections and relieve the pain and psychological burden of patients.

The main reason is that laser therapy mainly damages retinal oxygen-consuming cells through photocoagulation, reduces retinal thickness, and promotes choroid blood supply to the retina.^17^ The dilated capillaries are closed to inhibit the leakage, stimulate the retinal pigment epithelium cells, and accelerate the regression of edema. In addition, retinal pigment epithelium can damage adjacent hyperoxia-dependent photoreceptors after absorbing laser photocoagulation, improve the blood supply of the inner retina, and reduce the degree of hypoxia in the inner layer of the affected area.^17^ However, simply using laser therapy can affect the peripheral visual field to varying degrees, temporarily affecting vision, and making it difficult to effectively restore abnormal retinal vascular permeability. Therefore, the overall effect of the individual treatment is not satisfactory.^17^,^18^ Ranibizumab can directly act on VEGF, inhibiting endothelial cell proliferation and neovascularization. It can enter the affected eye through the retinal membrane, reducing vascular leakage by downregulating VEGF expression, thereby improving clinical symptoms in patients. Moreover, ranibizumab can also alleviate vascular occlusion.^19^,^20^ Therefore, laser therapy and ranibizumab can exert a synergistic effect. While intravitreal injection of ranibizumab can inhibit microvascular and capillary leakage, laser therapy can inhibit macular foveal extravasation. The combination of the two methods, thus, can synergistically eliminate edema, achieving the goal of improving patients’ visual function.^16^,^20^

In addition, studies have shown that VEGF is an important endogenous mediating factor in the pathogenesis and progression of DME, which can affect the tight junction protein in endothelial cells, damage the internal retinal barrier, cause vascular leakage, and then exacerbate the disease of DME.^8^,^21^ Nirtic Oxid Synthase (NOS) has vasoactive proprieties, and abnormal levels of NOS can lead to endothelial cell death and retinal blood flow reduction, thus causing local ischemia and hypoxia, and accelerating the occurrence and progress of ME.^21^,^22^ The results of our study showed that after the treatment, the serum NOS levels in the Combined-group were higher and the VEGF levels were lower than those in the Laser-group and Ranibizumab-group. These results further confirm the feasibility and effectiveness of the combined intervention regimen of ranibizumab and laser therapy in the treatment of DME. We may hypothesize that ranibizumab can act on VEGF receptor, preventing it from binding to endothelial cell surface receptor, inhibiting neovascularization, reducing vascular permeability and vascular leakage, reducing the degree of local hypoxia and ischemia, and reducing CMT. A combination of ranibizumab with laser therapy can have a synergistic effect and effectively improves patients’ vision and the overall treatment effect.^22^,^23^ In addition, we found that the incidence of complications of the combination therapy is lower than LP alone or Ranibizumab alone, which suggests that the combination therapy has a safe profile.

### Limitations

The study includes a small sample size and it is a retrospective study, which may introduce selection bias. Moreover, other indicators such as pigment epithelium-derived factor could be investigated as it is associated with vascular permeability in the eye and on the DME. Further multicenter prospective cohort study with larger sample sizes and additional indicators of macular morphology are needed.

## CONCLUSION

Compared to ranibizumab or LP alone, ranibizumab combined with LP can shorten the time of ME improvement, exudation absorption, and fundus hemorrhage absorption in patients with DME. In addition, the combined treatment can effectively regulates serum NOS and VEGF levels, improves patients’ vision, and reduces CMT and RNV.

### Authors’ contributions:

**JH:** Conceived and designed the study.

**HL**, **HX**, **YL** and **DY:** Collected the data and performed the analysis.

**JH:** Was involved in the writing of the manuscript and is responsible for the integrity of the study.

All authors have read and approved the final manuscript.
